# Maintaining Delivery of Evidence-Based Interventions to Reduce Under-5 Mortality During COVID-19 in Rwanda: Lessons Learned through Implementation Research

**DOI:** 10.5334/aogh.4348

**Published:** 2024-07-23

**Authors:** Alemayehu Amberbir, Felix Sayinzoga, Kedest Mathewos, Jovial Thomas Ntawukuriryayo, Amelia VanderZanden, Lisa R Hirschhorn, Agnes Binagwaho

**Affiliations:** 1 University of Global Health Equity, Kigali, Rwanda; 2 Maternal, Child, and Community Health Division, Rwanda Biomedical Center, Kigali, Rwanda; 3 Feinberg School of Medicine, Northwestern University, Chicago, IL, USA

**Keywords:** COVID-19, implementation research, under-5 mortality, essential health services, Rwanda

## Abstract

*Background:* The COVID-19 pandemic resulted in drops in access to and availability of a number of evidence-based interventions (EBIs) known to reduce under-5 mortality (U5M) across a wide range of countries, including Rwanda. We aimed to understand the strategies and contextual factors associated with preventing or mitigating drops nationally and subnationally, and the extent to which previous efforts to reduce U5M supported the maintenance of healthcare delivery.

*Methods:* We used a convergent mixed methods implementation science approach, guided by hybrid implementation research and resiliency frameworks. We triangulated data from three sources: desk review of available documents, existing routine data from the health management information system, and key informant interviews (KIIs). We analyzed quantitative data through scatter plots using interrupted time series analysis to describe changes in EBI access, uptake, and delivery. We used a Poisson regression model to estimate the impact of COVID-19 on health management information system indicators, adjusting for seasonality. We used thematic analysis of coded interviews to identify emerging patterns and themes.

*Results:* We found moderate 4% (IRR = 0.96; 95%CI: 0.93, 1.00) and 5% (IRR = 0.95; 95%CI: 0.92, 0.99) drops in pentavalent and rotavirus 2 doses vaccines administered, respectively. Nationally, there was a 5% drop in facility-based delivery (IRR = 0.95; 95%CI: 0.92, 0.99). Lockdown and movement restrictions and community and health-worker fear of COVID-19 were barriers to service delivery early in the pandemic. Key implementation strategies to prevent or respond to EBI drops included leveraging community-based healthcare delivery, data use for decision-making, mentorship and supervision, and use of digital platform.

*Conclusions:* While Rwanda had drops in some EBIs early in the pandemic, especially during the initial lockdown, this was rapidly identified, and response implemented. The resiliency of the health system was associated with the Rwandan health system’s ability to learn and adapt, encouraging a flexible response to fit the situation.

## Background

Early in the Coronavirus Disease 2019 (COVID-19) pandemic, policymakers and modelers warned of the threat to essential health services (EHS), including maternal and child health [1–3]. Over time, drops in access to and availability of a number of the EBIs known to reduce U5M were reported across a wide range of countries [1–3]. These drops were related both directly to the COVID-19 pandemic and to heath-system changes made in response to it.

Studies have also shown indirect effects of COVID-19 on child mortality in low- and middle-income countries [1, 2] related to changes in the availability and affordability of nutritious foods, as well as interruptions in healthcare and social protection services [3]. WHO’s Global Pulse Surveys conducted in May–July 2020 and January–March 2021 indicated 90% and 94% reductions, respectively, of EHS, with a reduction in the average percentage of disrupted services per country from 50% of 35 tracer services in 2020 to just over one-third in 2021 [4, 5]. Primary care services that required outreach, such as immunization, as well as rehabilitative and palliative care, saw the largest disruptions [4, 5].

The first Rwandan COVID-19 cases were reported on March 14, 2020, and immediate intervention measures, including contact tracing and lockdowns, were put in place. One week after the first case, the government imposed a complete national lockdown from March 21 to April 30, 2020, to minimize the virus spreading across the country, with preservation of essential services such as healthcare and food-related businesses [6, 7]. Throughout the pandemic, Rwanda implemented mask wearing, prohibition of mass gatherings, social distancing, temperature checks at airports and public buildings, vaccination, and the creation of separate COVID-19 treatment centers [8]. By July 2021, the country had registered over 86,242 COVID-19 cases, and 1.1% had died of the virus [9].

An assessment conducted by the Rwandan Biomedical Center (RBC) found variability in maternal and child health EHS maintenance [10]. The RBC reported maintenance of availability of services including antenatal care services at the health-center level, facility-based delivery provided in 97% of the health facilities, and continued delivery of other EHS for 97% of health facilities. In addition, 98% of health facilities maintained the provision of integrated management of childhood illness and immunization services and 99% continued to provide services to prevent mother-to-child transmission of HIV. A separate study using routine health management information system (HMIS) data also reported decreases in the utilization of maternal and child health services comparing March–April 2020 with the corresponding months in 2019. They found the utilization rate of facility-based delivery declined from 0.99 to 0.89 (*p* = 0.004), antenatal care 4th standard (ANC4) visit from 0.40 to 0.36 (*p* = 0.083), rotavirus 2 from 0.95 to 0.88 (*p* = 0.009), and pentavalent vaccine (diphtheria, tetanus, pertussis, hepatitis B, and haemophilus influenza type B) from 0.95 to 0.90 (*p* = 0.078) [11].

We had previously conducted an implementation research study of six “exemplar” countries, including Rwanda, to understand how these countries outperformed their regional and economic peers in reducing amenable U5M during the period 2000–2015. We applied a hybrid implementation science framework [12] to identify the implementation strategies and contextual factors that either facilitated or hindered progress in implementation of the EBIs. We found that the combination of strategies that were implemented at all steps and were owned at all levels of the health system allowed Rwanda to achieve significant implementation outcomes that ultimately resulted in a 74% reduction in U5M during the study period. It was initially feared that the COVID-19 pandemic would halt this progress and result in the interruption of health-service delivery.

Building on this case study work, we embarked on a study in Rwanda to explore if and how health-system-delivered EBIs targeting amenable U5M were maintained during the COVID-19 period and what strategies were used to prevent or respond to EBI drops; identify factors that helped or hindered the response to COVID-19-related threats to EBI uptake and delivery; and understand how the previous efforts to implement these EBIs between 2000 and 2015 supported the country’s work to maintain EBIs during the pandemic and contribute to resiliency.

## Methods

### Study design and approach

**Figure 1:**
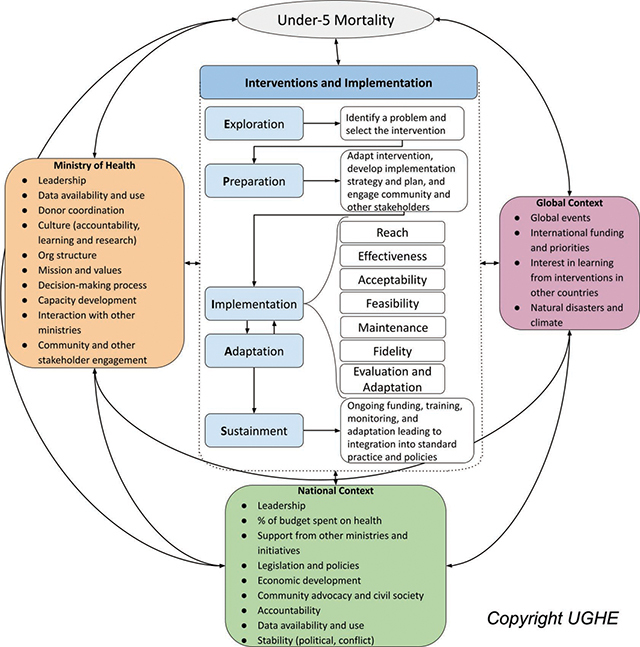
Implementation research framework for understanding evidence-based interventions to reduce under-5 mortality [12].

The study utilized a mixed methods implementation science approach. The work was guided by our implementation research framework designed as part of the exemplar work (Figure 1) [12]. This framework built on Aarons and colleagues’ Exploration, Preparation, Implementation, and Sustainment Framework (EPIS) [13], adding in a critical explicit step of Adaptation (EPIAS) [12]. We utilized this framework to understand strategies implemented and contextual factors experienced at the different levels, including global, national, Ministry of Health, subnational, facility, and community, during the COVID-19 pandemic period. We also used a health system resiliency framework adapted from Kruk and colleagues to explore evidence of health-system resiliency in Rwanda [14]. We explored the health-system-delivered EBIs found to directly reduce amenable U5M; identified strategies used, adapted, or newly adopted; and identified contextual barriers and facilitators responsible for either increased or decreased use and provision of EBIs known to reduce U5M.

### Data collection and measures

We collected data from three sources: desk review, existing routine data from the HMIS, and KIIs.

*Desk review:* Between March and June 2021, we extracted data from peer-reviewed publications accessed through PubMed and Medline; Ministry of Health policy documents and reports; and documents from WHO, Global Fund, Global Financing Facility, and other sources (see Appendix 1 for original search terms)**.** As feasible, we explored contextual factors and new strategies used by Rwanda and in the WHO region to maintain EBIs known to reduce U5M in the context of COVID-19.

*HMIS data:* To estimate coverage of the various EBIs known to reduce U5M during the initial period of COVID-19 in Rwanda, we analyzed existing, routinely collected national HMIS data with monthly counts aggregated at the district level for a given indicator. We specifically looked at administration of pentavalent and rotavirus 2 vaccines, ANC4, facility-based delivery, and cases of diarrhea and pneumonia treated at facilities as markers of essential health services targeting leading causes of U5M; we had previously used similar indicators in our earlier work [12]. We explored these data, disaggregated by geographic location and granular time unit, over the three years before COVID-19 (January 2017 to February 2020) and during the initial COVID-19 period (March to December 2020).

*Key informant interviews (KIIs):* We adapted the codebook from the previous study, based on emerging lessons from COVID-19 response and strategies associated with preventing, mitigating, or responding to drops in EBI access, uptake, and coverage, as well as Kruk and colleague’s resiliency frameworks [14]. KIIs were conducted with policymakers, donors, implementing partners, and direct health-services providers between February and April 2021 (Table 1). We designed the interviews to identify implementation strategies and how they were selected, adapted, or newly adopted. Key informants (KIs) helped identify additional evidence of implementation strategy success or lack thereof. In addition, the KIIs explored the process by which decision-making was made, as well as key contextual factors: barriers, facilitators, or other factors that influenced decision-making.

**Table 1:** Composition of key informants interviewed.

KEY INFORMANT REPRESENTATION	DEPARTMENT/ORGANIZATION
Ministry of Health – 9 (43%)	Clinical and public health services Planning and financing Administrative health units District hospitals Health centers
Rwanda Biomedical Center – 5 (24%)	High-level administration Case management unit Vaccine preventable diseases Community health Institute of HIV/AIDS Disease Prevention and Control
Implementing partner/donor – 4 (19%)	UNICEF US Agency for International Development (USAID) Non-governmental organizations (2)
Professional association – 1 (4.7%)	Rwanda Pediatric Association
Faith-based organization – 1 (4.7%)	Manager
Private sector – 1 (4.7%)	Pediatrician

### Data analysis

*Quantitative:* We analyzed quantitative data to describe changes in EBIs access, uptake, and delivery. We created scatter plots using interrupted time series analysis [15] (from January 2017 to December 2020). We used interrupted time series analyses by fitting a Poisson regression model to estimate the impact of COVID-19 on childhood immunization, maternal health services (antenatal care visits and facility-based delivery), and outpatient attendance for children. The model included a time variable, a dummy COVID-19 variable, and a primary health service reporting calendar month. We adjusted the model for seasonality using two pairs of sine and cosine terms (Fourier terms) included in the model. We analyzed the data using Stata 16.1 (Stata-corp, College Station Texas, USA).

*Qualitative:* We audio recorded, transcribed, translated where necessary, and entered the qualitative data from KIIs into Dedoose (v-9.0.17). We conducted an iterative coding in Dedoose and used code weighting to describe relative frequency of categories. We used thematic analysis of coded interviews to identify emerging patterns and themes from interviews [16].

We triangulated the desk review, HMIS, and KII data to do a quantitative-qualitative explanatory synthesis of if and how strategies mitigated or prevented drops, were adapted or newly implemented, and similar analysis for contextual factors.

## Results

### Desk review

**Figure 2:**
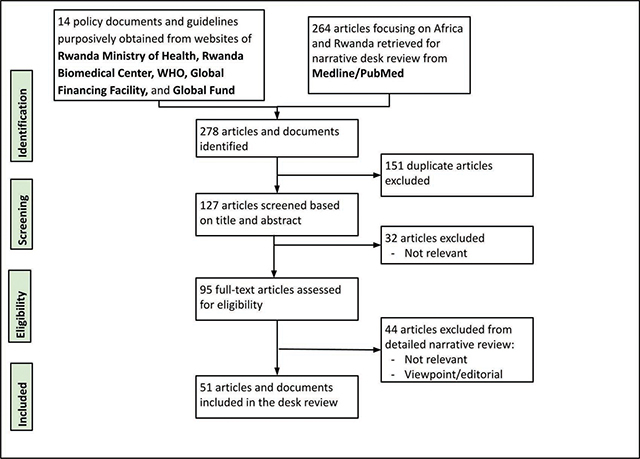
CONSORT diagram.

We identified a total of 264 Medline publications based on our search strategy related to COVID-19 situation and EHS service delivery in Rwanda and Africa, as well as 14 purposively identified policies and guidelines from the Rwanda Biomedical Center and global sources, including WHO. After elimination of 151 duplicates, 127 publications were screened based on title and abstract, and 51 articles and policy documents were included in the desk review after full text screening (Figure 2 shows the CONSORT diagram of study selection; details of individual study extraction are in Appendix 2).

*Potential disruption of EHS in Rwanda:* The Global Financing Facility used Lives Saved Tool (LiST) analysis to predict maintenance of EHS in Rwanda and found that there could be potential disruptions in access to oral antibiotics for pneumonia for 264,900 children, diphtheria-pertussis-tetanus (DPT) vaccinations for 459,900 children, and access to facility-based delivery services for 93,300 women in Rwanda. This analysis warned that these disruptions could then lead to an increase in child mortality by 29%, and maternal mortality by 23%, by the end of 2021 [17]. By contrast, the RBC conducted a rapid assessment and found that while there was an overall decrease in new antenatal care attendance in 2020 as opposed to 2019, there was a slight increase in antenatal care attendance from March to May 2020 and maintenance of delivering antenatal care services at the health-center level, with facility-based delivery provided in 97% of the country’s health facilities [10].

*Response strategies:* In the desk review, we found that countries adopted various strategies to mitigate or respond to the drops in coverage of EHS. These included defining a list of EHS that needed to be maintained, allocating a focal person to this task, and designating funding to maintain these services [5]. Countries leveraged data availability and use to monitor and track information to support EHS and implement mitigation strategies accordingly. Countries also monitored implementation of strategies to mitigate disruption. Health facilities recruited additional staff, reassigned existing staff, and adopted digital platforms to replace in-person consultations with telemedicine. In some cases, the delivery of services was adapted. For instance, some HIV/AIDS patients received extended drug prescriptions [5]. At health facilities, cases were triaged to identify priorities and some patients were redirected to alternate sites. To increase health-seeking behavior and engage with the community, health facilities used community communications methods.

### Interruptions in EBI delivery (HMIS)

**Figure 3 a and b:**
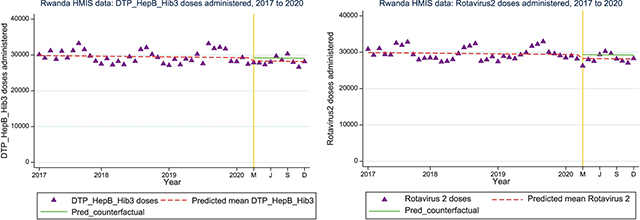
Interrupted time series analysis for pentavalent (DPT/HepB/Hib3) and rotavirus 2 vaccine doses administered nationally from 2017 to 2020.

*Routine immunization*: Nationally, interrupted time series analysis for doses of pentavalent (IRR=0.96; 95%CI: 0.93, 1.00) and for rotavirus 2 (IRR=0.95; 95%CI: 0.92, 0.99) vaccination administered showed a 4% and 5% drop during the initial period of the pandemic respectively (Figure 3).

**Figure 4 a and b:**
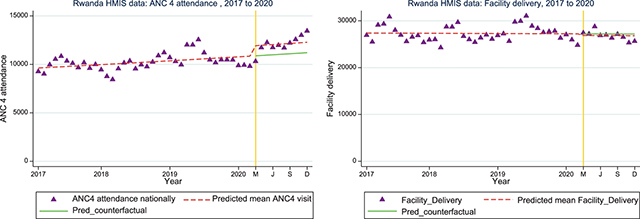
Interrupted time series analysis for ANC4 attendance and facility-based delivery nationally.

*ANC4 attendance visits and facility-based delivery:* Nationally, there was a slight increase in ANC4 visits during the initial period of COVID-19 (March to December 2020) compared with the existing trend prior to COVID-19 (January 2017 to February 2020) (IRR=1.07; 95%CI: 1.01, 1.14). Facility-based delivery dropped by 5% (IRR=0.95; 95%CI: 0.92, 0.99) nationally (Figure 4).

**Figure 5 a and b:**
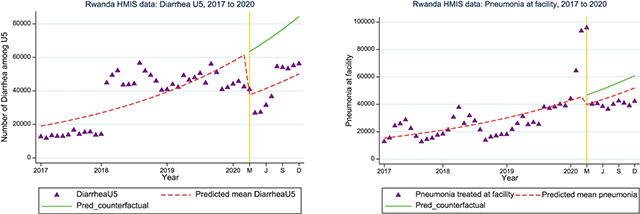
Interrupted time series analysis for diarrheal and pneumonia cases treated at facility nationally.

*Diarrheal and pneumonia cases treated at facility:* HMIS data comparing three years of trends on diarrheal and pneumonia cases treated at facility and community showed marked drops in both diarrhea cases (diarrheal cases treated at facility [IRR=0.58; 95%CI: 0.43, 0.79]) and pneumonia cases treated at facility (IRR = 0.85; 95% CI: 0.65, 1.11) during the first nine months of COVID-19 pandemic (Figure 5). When asked about this disruption, KIs explained that the COVID-19 prevention measures such as handwashing, social distancing, and school closures reduced the transmission of these diseases as well.

### Results of KIIS

We interviewed a total of 21 KIs in the study. KIs were selected to represent all stakeholders within the health system, including Ministry of Health (43%), Rwandan Biomedical Center (24%), implementing partners or donors (19%), professional association (4.7%), faith-based organization (4.7%), and private sector (4.7%) (Table 1). These included policymakers, managers, implementers, and clinicians.

*Transient reduction in the delivery of EHS:* The KIs who reported observing disruptions reported the following services to be interrupted: vaccine coverage, outpatient department consultations for non-severe illnesses, antenatal care visits, facility-based delivery, growth monitoring, and missed appointments for noncommunicable disease patients. The other EBIs we considered in the study were not reported to have been interrupted. These disruptions were largely reported between mid-March and early May 2020, which was the period of Rwanda’s first COVID-19 lockdown. Overall, according to a majority of the KIs, the reduction was neither severe nor prolonged; there were a few, however, who did consider it to be severe.

### Contextual factors

The facilitating contextual factors identified during the desk review and in the KIIs reflected very closely what was identified during the first study, which looked at the period 2000–2015. Rwanda’s strong primary healthcare system, accountability of the leadership, and strong community health system are some examples of facilitating contextual factors that helped Rwanda reduce U5M between 2000 and 2015 and also contributed to Rwanda’s efforts to mitigate or respond to drops in EHS coverage during the COVID-19 pandemic. However, the challenging contextual factors that hindered the maintenance of EHS were new: factors such as lockdown and fear of COVID-19 were new and largely related to the spread of the virus itself and the response to the pandemic. We describe the contribution of these factors to the maintenance of EHS below as identified in the KIIs. Table 2 outlines these contextual factors and the mechanism through which they either facilitated or hindered health-service delivery.

**Table 2:** Contextual factors identified from the key informant interviews.

TYPE	CONTEXTUAL FACTOR	STATUS *E=EXISTING N=NEW*	KEY TAKEAWAYS
Facilitating Contextual Factors	Culture of collaboration and coordination (donors, between sectors)	E	Existing structure and culture of collaboration between government/ implementing partners/donors/technical working groups/professional associations through the national strategic plan prevents duplication Facilitates accurate decision-making by allowing for a holistic view
	Strong preexisting community health system and structure, including community health workers	E	Strong facilitator of community level childcare and follow up as community health workers live within the communities Responsible for communicating the continuation of health services to prevent stops in access (although some were overburdened during the pandemic)
	System of learning and improvement	E	Regular monitoring and evaluation supported identification of disrupted health services and informed decision-making at all levels Continuous learning from global and local data Ability and willingness to adapt based on emerging data
	Preexisting culture and capacity of data use	E	Availability of data through HMIS allows the Ministry of Health to follow the trend of service coverage during the pandemic to inform and facilitate the systems of learning and improvement Centralized data reporting allows the government to make decisions with a holistic view of the situation in the country
	Health-system design decentralized and focusing on primary healthcare	E	The decentralized nature of the healthcare system increased access to health services before and during the pandemic Use of separate COVID-19 treatment centers prevented cross-infection in hospitals and contributed to reduction of fear The existence of one system instead of multiple, parallel systems prevents competition and allows the Ministry of Health to reach the whole population
	Strong supply-chain system	E	Strong supply chain and stock of drugs that prevented stock-out of drugs and so reduced a cause of delivery interruption Ability of the supply chain system to adapt to arising challenges (e.g., push rather than pull system)
	Strong leadership and control	E	Strong, centralized leadership that guides the involvement of donors and implementing partners Leadership committed to investing in the health and well-being of its population
	Health-system structure	E	Strong inputs into the system that supported the continuation of health services: e.g., Mutuelle de Santé, strengthened healthcare capacity through HRH, cascade training/mentorship structure Guidance provided from technical working groups and professional associations
	National and local ownership and authority of the health system and EBI delivery	E	Rwanda’s culture of coordination of partners with strong leadership at the central and local levels allowed the country to guide donor and implementing partners’ funding and project priorities towards the needs of the country Despite fear, healthcare workers were committed to providing care
	Culture of accountability	E	Commitment to serve the vulnerable, critical during COVID-19 as the vulnerable were most affected Accountability beyond the health sector including the political leadership at district levels, e.g., *imihigo* contracts Resulting trust increased adherence to public health measures and continued uptake of health services during the pandemic
	Organizational culture and climate	E	The system was designed to adapt to the needs and behaviors of the population
	Resilient health system due to previous epidemic preparedness (e.g., Ebola virus)	E	The system that was built prior to the pandemic prepared the country to respond to COVID-19 Facilitating contextual factors such as strong leadership and the culture of collaboration existed prior to the pandemic Many of the strategies such as community education and engagement existed prior to COVID-19 and support mitigated EBI drop
Challenging Contextual Factors	Lockdown/movement restriction	N	Lack of public transportation hindered population access to healthcare services and healthcare workers’ ability to reach their facilities Increased costs of service delivery as adaptations were made to respond to this challenge Challenged regular supervision and monitoring, shifting to virtual meetings
	Fear of COVID-19 by the community	N	Fear of infection at facilities tackled through health communication Report of community fear varied by district—regions with more COVID-19 cases more likely to report community fear of COVID-19
	Fear of COVID-19 by healthcare workers	N	Novelty of the virus and lack of personal protective equipment contributed to healthcare workers’ fear of COVID-19 Training on COVID-19, reassurance from leaders and realization that COVID-19 patients were surviving helped prevent/address the fear of infection
	Workload/staff shortage	N	Healthcare workers moved from their usual positions to COVID-19 treatment centers Healthcare workers, overwhelmed with additional responsibility, received support from the government and partners
	Stock-outs	N	Overall, stock-out was not a challenge in Rwanda Country prepared with one-year stock of vaccines and drugs

CHW: community health worker; EBI: evidence-based intervention; EHS: essential health services; HMIS: health management information system; HRH: human resources for health; KI: key informant; KII: key informant interview; U5M: under-5 mortality

### Implementation strategies

Rwanda implemented various strategies to prevent or respond to health-system-delivered EBIs toward essential maternal and child health interventions. Most of these strategies existed pre–COVID-19, but Rwanda implemented them with variable levels of adaptation (Table 3). We describe three of these key strategies below.

**Table 3:** Implementation strategies used to prevent or respond to drops in evidence-based interventions.

STRATEGIES	PREVENT AND/OR RESPOND TO EBI DROP	NEW (N), ADAPTED (A), OR CONTINUED (C) STRATEGY	KEY TAKEAWAYS *A= ADAPTED; C= CONTINUED; N=NEW; E= EXISTING*
Mentorship and supervision	X	A, N	Technical mentorship using pediatricians to monitor neonatal care at districts to continue core activities during COVID-19 (A) Supervision of community health workers via telephone as a response to COVID-related challenges to in-person monitoring and supervision (A) Following COVID-19 prevention measures—e.g. limiting no. of supervisors per vehicle (N)
Data use	X	A, C	Use of data at the lower level of care including Data Quality Assessment, maternal death audit for local decision-making (E) Partners use the same HMIS data platform, analyze and make decisions at coordination meetings and technical working groups (E) Use of electronic register for vaccination to help track missed children (E)
Community engagement/ education	X	A, C	Use of radio and public television to spread the right information, e.g., using community health workers, local leaders and church leaders (A), SMS message reminders to mothers in the vaccine program (N), integrated risk communication in all existing community interventions (A) Educate the community to avoid fear, encourage health-seeking behavior, and facilitate transportation for patients (A)
Enacting policies to support essential health services maintenance	X	A, C, N	Policy prioritizing essential healthcare services during COVID-19 (A) “Imihigo” or leadership performance contracts—ensures accountability at all levels (E) Directives from the ministry of health for health workers to cancel vacations to avoid staff shortage (N) Staff who lived far from a health center had to relocate and move to a rental house closer to the health facility (N)
Provision of transport	X	A, N	Provision of transport to patients and healthcare providers particularly during the lockdown (government + partners) (N) Provision of vehicles and motorbikes to health centers to support vaccination programs (E)
Leveraging existing systems	X	A, C	Cross-Sectoral National Joint Task Force was established before the first case of COVID-19 based on experience with preparedness for Ebola (A) Ebola preparedness and availability of personal protective equipment for COVID-19 response (N)
Supply-chain strengthening	X	A, C	Planning well in advance of shipment of supplies to avoid stock-out Redesigning the supply chain system for vaccines using push system, e.g., active distribution of vaccines and supplies to districts using refrigerator truck. Using local manufacturers due to international disruption
Donor and implementing partner coordination	X	A, C	Partner coordination meetings happening regularly using virtual platform (A) All technical working group meetings continued to work virtually during COVID-19 and ensure continuity of EHS and clinical services (A)
Focus on equity	X	A, C, N	Data analysis by geography to see if there is any equity gap (e.g., districts with low coverage supported) (E) Redirect money from programs to support vulnerable population (informal sector) (N) Support vulnerable groups during the lockdown (e.g., nutritional support) (N)
Community-based healthcare delivery	X	A, C	Using community health workers to identify children who missed vaccination; conduct house-to-house growth monitoring, vitamin, and deworming distribution; visiting schools; and organizing outreach campaigns (A) Emphasis on primary healthcare and community-based health insurance—affordable health services (E)
Digital platform	X	A, C, N	WhatsApp groups with data managers, community health workers, and supervisors for data audit, discuss M&E activities, and maintain coordination (A) WhatsApp group among professional members to discuss activities and exchange new information (E) Webex and zoom virtual meetings with partners and stakeholders (A) Remote mentorship using simulation-based training (less robust) and mobile phone consultations (A)
Response to COVID-19 and support to maintain evidence-based interventions	X	A, N	Safety: community health workers and health facilities were provided with personal protective equipment and other COVID-19 prevention measures; this supported EHS maintenance Education: Toll-free number specific to COVID-19, also providing other EHS Coordination: Establishment of command posts from national—the National Joint Task Force—to cell level to ensure all the coordination around COVID-19 runs smoothly Health system support: Support logistics of COVID-19 response, information-sharing across the country and community engagement Change in delivery: Additional under-5 vaccination days to respect social distancing and prevent spread of COVID-19 but also maintain vaccination
Human resources strengthening	X	A, C	Reallocation of funds for the recruitment of new staff and volunteers (A) Staff support from partners to ensure avoid pulling out of many healthcare providers from their normal activities to COVID-19 treatment centers (A) Restructuring of staff from health centers to health post to increase outreach services for particularly for vaccination (A)

CHW: community health worker; EBI: evidence-based intervention; EHS: essential health services; HMIS: health management information system; KI: key informant; KII: key informant interview; U5M: under-5 mortality

#### Leverage community-based healthcare delivery (existing)

Rwanda’s community health system has been celebrated for its contribution to the country’s rapid improvement in maternal and child health outcomes in recent decades. Specific implementation strategies related to leveraging community-based healthcare delivery included using community health workers (CHWs) to identify children who missed vaccination. CHWs created links between the community to the health facility, organized community outreach programs, educated community members to avoid fear and encourage health-seeking behavior, directed patients who were sick and facilitated transportation, made home visits to newborns, conducted house-to-house growth monitoring as well as vitamin and deworming distribution, and organized outreach campaigns. These strategies were undertaken with variable adaptations. Other implementation strategies related to community-based healthcare delivery which existed before COVID-19 include Rwanda’s strong emphasis on primary healthcare and community-based health insurance, which provided affordable health services closer to the community, including free services related to COVID-19 as part of the *mutuelles*, or community-based health insurance, scheme. A KI from a community health program explained,

*“As people were in their homes, with their families, there was no movement and…we did a close monitoring of communities because CHWs were working together daily with all the families. Even today we are still in pandemic, they are the one who are giving vitamins and deworming since Monday up to the 7th of March.”*

#### Data use for decision-making (existing)

Rwanda’s system of learning and use of data for decision-making were key strengths of the resilient health system. Many KIs emphasized the value of using routine health-facility data throughout the pandemic to identify changes in EBIs and implement interventions. Specific use of this strategy during the period of COVID-19 to respond to or prevent EBI drops included use of data at the lower level of care for early identification of changes in EBIs, monthly data review and quality assessment meetings at the health-center level to inform decisions, age disaggregation of data to identify risk groups, maternal death audit for local decision-making, partners’ use of the same HMIS data platform, and utilization of electronic registers for vaccination to help track missed children. One KI said,

*“Data played a big role, especially in the response [to COVID-19] for two reasons. Number one, data has helped us to shape the response. So as we keep using data going forward it helps us to shape the response, to see how the trends are decreasing or increasing. And then it helps us to adapt some measures.”*

Another KI explained,

*“Normally each month, we analyze data from the CHWs’ report, and when we had partners meeting, we present this data and see the situation. Because each partners have specifically assigned districts, they need to discuss with the government which strategy to use to mitigate a problem. For example, in a given district, we may experience an increase in death, a strategy will be designed and tested to see if this can also be shared to other partners. This is a monthly monitoring meeting, give feedback during the next TWGs [technical working groups] meetings.”*

Rwanda further utilized its capacity for being aware of the status, delivery, and challenges of healthcare interventions building on data use, strengthened data systems, and regular review and action associated with accountability. Rwanda was aware of the potential disruption of health services during the COVID-19 pandemic elsewhere and prepared accordingly to prevent, mitigate, and respond to drops. As a KI explained, “We are a bit prepared and the other all stakeholders ready, a government policy, the government will, support and stakeholders from the top down and down to the top.”

#### Mentorship and supervision (existing/adapted)

Mentorship and supervision were important implementation strategies utilized with some level of adaptation to COVID-19 preventive measures to ensure continuity and delivery of quality EBIs. Most KIs reported a disruption and delay in conducting regular in-person district-level supervision and meetings due to COVID-19-related measures and transport restrictions. Most supervision activities were moved to online platforms, with some in-person district-level supervision as was feasible. Specific implementation strategies included conducting technical mentorship using pediatricians to monitor neonatal care (e.g., neonatal resuscitation and management of hypothermia) at districts to continue core activities during COVID-19. CHWs received supervision via telephone as a response to COVID-related challenges to in-person monitoring and supervision as an adapted strategy, and adaptation of the in-person supervision following COVID-19 prevention measures included limits on the number of supervisors per vehicle travelling to districts. A KI also explained,

*“For example for mentorship, we have a mentorship program where we trained some district based mentors at hospital level to mentor the health center…we continued to support them remotely for example over the phone and WhatsApp calls and so on, so they could continue. Because within the district people could continue to support those activities, so we continued to do that online.”*

### Contribution of rwanda’s effective response to COVID-19 to the maintenance of EHS

An effective COVID-19 response mitigated the impact of the pandemic and helped the country maintain the delivery of EHS by preventing the healthcare system from being overwhelmed by a surge in COVID-19 infections. Specific strategies in the response to COVID-19 included provision of CHWs and health facilities with personal protective equipment and other COVID-19 prevention measures which supported the maintenance of EHS, the establishment of a toll-free number to provide COVID-19 specific education and community education to avoid fear, and provision of other services including laboratory tests, as well as providing information to other EHS. Rwanda established command posts from the national level (e.g., the National Joint Task Force) to cell level (the lowest administrative unit with a command post) to ensure coordination around COVID-19 ran smoothly. This also supported the maintenance of EHS, the logistics of the COVID-19 response, information-sharing across the country, and community engagement.

One KI explained,

*“Command posts have been put [in place] to ensure all the coordination around COVID-19, that means especially going back to that, the command post played the role in the coordination of the logistics, ensuring the availability of cars, transport for the people to get services especially people moving from one place to another because it is a multi-sectoral body with different people in the command post. It played a big role in logistics and in information-sharing across the country to ensure people were really getting services.”*

### Implementation outcomes

Different implementation strategies adapted or newly implemented during the pandemic were associated with implementation outcomes, including acceptability, equity, reach or coverage, appropriateness, fidelity, feasibility, and effectiveness. KIs reported that there was either no change or a slight reduction of EHS delivery in the early months of COVID-19, with some subnational variability. For example, funding for these services was not reduced or redirected to COVID-19 prevention and response, reflecting fidelity. However, childhood immunization coverage was found to be lower in some districts (below 70%) and high in others (above 80%), reflecting subnational inequity in the vaccination coverage.

### Evidence of health system resilience

A critical theme that emerged from the KIIs and other data was the resiliency of the Rwandan healthcare system during the COVID-19 pandemic. The system that was built prior to the pandemic to respond to the everyday health needs of the Rwandan population adequately prepared the country to respond to COVID-19 and enabled it to prevent and mitigate the impact of COVID-19 and the resulting response on the delivery of ordinary health services. Facilitating contextual factors such as strong leadership and a culture of collaboration existed prior to the pandemic and facilitated the maintenance of EHS. For instance, the culture of collaboration allowed different sectors and external stakeholders to work together to respond to COVID-19 and to maintain the delivery of EHS. Similarly, many of the strategies such as community education and engagement existed prior to COVID-19 and allowed the government to encourage health-seeking behavior during the pandemic. These strategies, most of which were existing or adapted, also allowed the country to respond to emerging challenging contextual factors such as fear of COVID-19 infection and movement restrictions. Table 4 describes key takeaways on health-system resiliency according to the five domains adopted and adapted from the original framework developed by Kruk et al. [14].

**Table 4:** Strategies and factors mapped on health system resilience.

	DOMAIN	KEY TAKEAWAYS
1	Adaptive	Strong motivation to adapt strategies to avoid negative health outcomes Adaptation of service delivery to reach patients while abiding by COVID-19 guidelines Task shifting and reallocation of budget to bolster healthcare worker capacity for COVID-19 response and to maintain EBIs
2	Aware	Aware of and well prepared for a potential disruption of health services during the pandemic Awareness of the virus, protection methods, and trainings reduced fear among healthcare workers Aware of drops in EBIs through the regular evaluation of data
3	Self-regulating	Maintained core function through the prioritization of health services Critical follow-up of patients with other diseases
4	Integrated	Maintained straight lines of communication and referral between the different levels of the healthcare system Coordination with trusted non-health actors for health communication
5	Diverse	All service delivery activities continued with the support of leadership despite the threat of COVID-19 Multiple approach for EHS delivery

### Transferable lessons

Several lessons emerged from Rwanda which would be relevant for other countries aiming to prevent or respond to a drop in health-system-delivered EBIs for the reduction of U5M during the period of COVID-19, or to build resiliency for future health threats. Key transferable lessons include investing in health systems, inputs and quality; leadership at all levels; coordination of activities; data use for decision-making; ownership and accountability; timely information and communication; and ensuring public trust. Many of these lessons emerged in the period of Rwanda’s work to address U5M between 2000 and 2015 and were further emphasized in response to the COVID-19 pandemic.

## Discussion

This mixed methods implementation research study found that Rwanda, despite some level of disruption and drop in access during the early months of the COVID-19 pandemic, largely maintained the EBIs for the reduction of U5M. The HMIS data analysis showed that there were 4–5% drops in pentavalent and rotavirus 2 vaccines and facility-based delivery uptake in 2020 as compared to 2017–2019. Diarrheal and pneumonia cases treated at facility and community showed a marked drop (15% to 40% reduction) during the initial period of COVID-19. KIs explained that the COVID-19 prevention measures reduced the transmission of these diseases as well.

Consistent with the changes observed from the HMIS analyses and desk review, most of the KIs from different levels of the health system reported minimal disruptions of EHS overall during this period. The services that KIs reported had transient disruption included vaccine coverage, outpatient department consultations for non-severe illnesses, antenatal care visits, facility-based delivery, growth monitoring, and missed appointments for noncommunicable-disease patients. These disruptions were largely reported from mid-March to early May 2020, which was the period of Rwanda’s first COVID-19 lockdown. While there were reports of disruptions, a majority of the KIs agreed that delivery of EHS returned to normal levels soon after the lockdown was lifted. KIs reported that community members changed their location of health consultations depending on their knowledge of COVID-19 and their fear of infection.

Our study explored various reasons for the disruptions in EBIs, particularly during the initial phases of the pandemic, and the strategies related to responding. The factors described in the KIIs largely concurred with those reported globally [4, 5]. For instance, in both WHO Pulse Survey reports, countries noted reduced outpatient care attendance, reduced access to healthcare services due to lack of transport during lockdowns, financial difficulties, and fear as challenges [4, 5]. On the supply side, some countries reported cancellation and unavailability of services, overburdening of healthcare staff capacity due to redeployment to COVID-19 relief, insufficient personal protective equipment for healthcare workers, and supply-chain difficulties. While Rwanda also faced these challenges, they were not severe enough to disrupt EHS for an extended period of time.

This study found a number of implementation strategies and contextual factors (both barriers and enabling factors) that were associated with preventing or mitigating drops in the delivery of EBIs known to reduce U5M. The facilitating contextual factors identified during this study reflected very closely what was identified during the study looking at the period of 2000–2015 [18]. Rwanda’s strong health system (particularly at the primary-care level), the accountability of the leadership, the community health system, the strong supply-chain system which prevented stock-outs, and the robust health information system are some examples of facilitating contextual factors that helped Rwanda reduce U5M between 2000 and 2015. These are also some of the same factors that contributed to Rwanda’s efforts to mitigate or respond to drops in EHS coverage during the COVID-19 pandemic. Moreover, Rwanda had already developed a preparedness and response plan for a potential pandemic like COVID-19, based on experiences preventing an outbreak of Ebola virus disease, which had surged in West Africa in 2014 and the Democratic Republic of Congo in 2018 [19], as well as the H1N1 influenza pandemic in 2009 [20]. By contrast, the challenging contextual factors that hindered the maintenance of EHS were newly identified. Factors such as lockdown, which resulted in a lack of transport and increased financial vulnerability, fear of COVID-19, and workload/staff shortage were new and were largely related to the spread of the virus itself and the response to the pandemic.

Rwanda implemented a number of existing and new or adapted strategies to prevent or respond to EBI drops while responding to the COVID-19 pandemic. While most of the implementation strategies existed prior to the COVID-19 period, Rwanda implemented them with variable levels of adaptations. The rapid and successful response to the COVID-19 pandemic included the establishment of a strong, decentralized command post to lower administrative units to support coordination of logistics and provision of personal protective equipment, to ultimately ensure the delivery of EHS at facilities and in the community. Rwanda also leveraged its community-based healthcare delivery system to conduct various community activities and emphasize its primary healthcare and community-based health insurance, which provided affordable health services closer to the community. Rwanda’s use of data for decision-making and its existing system of learning were key strengths of the resilient health system to rapidly identify, within a month, gaps in service delivery. Provision of transport to patients and healthcare providers particularly during the lockdown supported movement to and from health facilities. Effective utilization of various digital platforms and the strong line of communication between local and central levels supported the continuation of EHS.

Countries adopted various strategies to mitigate or respond to the drops in coverage of EHS [5]. As in Rwanda, countries leveraged data availability and use to monitor and track information to support EHS and implement mitigation strategies accordingly; health facilities recruited additional staff, reassigned existing staff, and adopted digital platforms to replace in-person consultations with telemedicine [5]. These strategies are also very closely aligned with the strategies recommended by the WHO under its operational guidance on the maintenance of EHS provided in March 2020 [5].

In the past, health crises such as the 2014 Ebola outbreak disrupted the delivery of maternal and child health services,and many called for resiliency as a core component of functional health systems [21]. Kruk and colleagues argue that resilience emphasizes “functions health systems need to respond and adapt to health shocks, introducing a dynamic dimension into more static health system models which can help the system cope with surges in demand and adapt to changing epidemiology and population expectations of care” [14]. Due to the existing broader facilitating contextual factors such as strong leadership, culture of accountability, as well as decentralization and supply-chain and information systems, Rwanda’s health system was able to continue providing EHS for children under 5, despite the shock of COVID-19. The strategies used to deliver healthcare services to children under 5 prior to the pandemic were used during the crisis to continue delivering EHS for children. The adaptability of these existing strategies such as community engagement, both to respond to COVID-19 and to maintain the delivery of EHS, is evidence of the dynamic nature of the health system and consequently its resiliency in the face of health-system threats.

Rwanda’s first case of COVID-19 was reported on March 14, 2020, and the country immediately put in place intervention measures, including contact tracing and lockdowns [22]. It is not likely that the spike in pneumonia/acute respiratory infection in the first months of 2020 shown in Figure 5 is due to COVID-19 cases, as there is little evidence of the virus circulating in Africa during this period; however, we cannot completely rule out the presence of any circulating acute respiratory infection, including potential issues with seasonality or issues with reporting.

This study must be interpreted in light of its limitations. Representation from community members, patients, and/or care seekers in our KIIs was not possible due to time and resources constraints, and would have provided valuable insight from the demand side. The ability to detect transient changes in EHS delivery and coverage is limited by the shorter duration of the period of COVID-19 under investigation. We did not include analysis of subnational variability of EBI delivery. Work to understand change over longer time periods and subnational variability will complement this study.

## Conclusions

This study found that while Rwanda had a drop in some EBIs during the early phase of the pandemic, especially during the initial lockdown, the country rapidly identified drops and implemented a response. As a result, Rwanda was able to continue most of the EBIs for the reduction of U5M during the first nine months of COVID-19. Lockdown, movement and transport restrictions, and fear of COVID-19 by the community and healthcare workers were important barriers to service delivery. Key implementation strategies used to prevent or respond to EBI drop included effective response to COVID-19, leveraging community-based healthcare delivery, data use for decision-making, mentorship and supervision, provision of transport, community engagement and education, and use of digital platforms. The resiliency of the health system was due to previous work to strengthen health-service delivery as well as the ability of the Rwandan health system to adapt, which encouraged a flexible response to fit the situation.

## Declarations

### Data availability

The data that support the findings of this study are available from the Rwandan Ministry of Health, but restrictions apply to the availability of these data, which were used for the current study, and so are not publicly available. The datasets used and/or analyzed during the current study are available from the corresponding author on reasonable request and with permission of the Rwandan Ministry of Health.

## Additional File

The additional file for this article can be found as follows:

Supplementary material. Appendix. Desk review results.
