# Supplementary material for: Maintaining Delivery of Evidence-Based Interventions to Reduce Under-5 Mortality During COVID-19 in Rwanda: Lessons Learned through Implementation Research
Source: Ann Glob Health. 2024 Jul 23;90(1):47. doi: 10.5334/aogh.4348 (PMC11276474; doi:10.5334/aogh.4348)
Supplement: Supplementary material. — Appendix. Desk review results. [file agh-90-1-4348-s1.pdf]

Appendix 1. Desk review search terms

We performed initial searches through MEDLINE (PubMed), World Health Organization (WHO) Global Health Library, Google Scholar, and other websites collecting publications on COVID-19 combination of search terms "COVID-19 AND Rwanda," "Child mortality AND COVID-19 AND Rwanda," "Child mortality AND COVID-19 and Africa," "Maternal-mortality AND COVID-19 and Rwanda," "Malaria AND COVID-19 and Rwanda," "ARI AND COVID-19 and Rwanda", "Immunization AND COVID-19 and Rwanda", "Pneumonia AND COVID-19 and Rwanda", "Disruption AND under-5 mortality AND COVID-19 AND Rwanda", "Healthcare AND COVID-19 AND Rwanda", "Health Service AND COVID-19 AND Rwanda "and specific EBIs and implementation strategies identified during the initial exemplar work. We also reviewed gray literature which included Rwandan situation reports for COVID-19 and COVID-19 government response guidelines and reports, as well as other sources including WHO guidelines, WHO pulse surveys, and World Bank reports.

Appendix 2: Desk review of Rwandan MOH report and policy documents

Document types: Journal article, Policy document, Guidelines, Report, Bulletin article, Plans, Public notices

Key findings: EBI's addressed, implementation strategies (including emerging strategies), contextual factors (including barriers/facilitators), implementation outcomes (Coverage/reach, acceptability, feasibility, effectiveness, equity, fidelity)

#	Author	Title	Document type	Key findings
Purposively identified Rwandan MOH reports and policy documents				
1	Rwanda Biomedical Center	Rapid Assessment for Continuity of Minimum Essential Reproductive Maternal, Newborn, Child and Adolescent Health and Nutrition services in the Context of COVID-19 Response in Rwanda.	Assessment report	The assessment evaluated availability and use of EHS: RMNCAH services including HR, essential drugs and supplies, as well as gaps and potential of COVID on the EHS. Key findings:<br>• Overall decrease in new ANC attendance in 2020 as opposed to 2019, a slight increase in the ANC attendance from March-May 2020.<br>• All HCs continued to provide ANC services<br>• 3% of health facilities received additional financial support for RMNCAH.<br>• 89% of health facilities reported using guidelines for maintaining delivery of RMNCAH.<br>• 40% of health facilities reorganized the services due to COVID-19.<br>• 97% of health facilities continued to provide delivery services.<br>• Stockout of PPEs, Syringes, antibiotics, gloves reported in March-May 2020.<br>• WASH services were generally available in all HFs, but in 30%, the services were either lacking or inadequate.
2	Ntazimira C, Birindabagabo P, and Mbituyumuremyi A	The Impact of COVID 19 on Malaria Program in Rwanda: Rapid Assessment of Knowledge, Attitudes, Perspectives on	Assessment report	Evaluation of Community Health Workers' and healthcare providers' knowledge, attitude, and practices about Covid-19, as well as barriers related to malaria services due to as Covid-19 mitigations measures. Key findings:<br>• Malaria services continued to be offered<br>• 73.9 of health workers and 56.5% of CHWs had appropriate COVID19-related knowledge

#	Author	Title	Document type	Key findings
		COVID 19, and Situation of Malaria Services Provision in Three Districts in Rwanda.		• Transport to health facilities due to movement restriction was the most common barrier.
3	UNICEF	Rwanda COVID-19 situation report n° 06.	Report	Situation report aimed to provide situation status of COVID-19 and response measures implemented in Rwanda.<br>• UNICEF provided more than 3,000 hand sanitizer bottles of 500ml for CHWs<br>• UNICEF provided 3 tons of chlorine powder and 1,500 liters of hand sanitizer to 22 hospitals
4	Hartnett BNJ, Nsanzabaganwa C, et al.	COVID-19 Rwanda response updates March 14 – April 25, 2020.	Bulletin article	This report highlights strategies for COVID response:<br>• Health education on COVID-19 prevention and response<br>• Screening at 31 points: 1 airport and 30 land and water entry points<br>• Testing at national reference laboratory capacity in place since early February 2020.<br>• Risk communication and community engagement through national awareness campaigns<br>• Logistics: quarantine sites, building health facility capacities with isolation units
5	Rwanda Biomedical Center & Rwanda Ministry of Health	Standard Operating Procedures for Preparedness and Response to COVID-19 outbreak.	Guidelines	Early March 2020, development of SOPs on:<br>• COVID-19 surveillance<br>• Clinical specimen (sample) collection, handling, storage and transport for COVID suspect cases<br>• Infection Prevention and Control (IPC)<br>• COVID-19 case management<br>• Psychological support<br>• Risk communication, community mobilization, data management
6	Rwanda Biomedical Center & Rwanda Ministry of Health	Coronavirus disease 2019, National preparedness and response plan.	Plan	• Implementation strategies for COVID-19 (before emergence of the 1st case)<br>• Preparedness budget by thematic area<br>• COVID-19 response plan<br>• COVID-19 response budget by thematic area<br>• Monitoring and evaluation framework<br>• Continuation of EHS (HIV, TB, malaria, mental health, NCDs, and MCH services) and protection of healthcare workers and patients
7	Rwanda Biomedical Center & Rwanda	COVID-19 clinical Management guidelines.	Guidelines	• Management of contacts<br>• Laboratory testing<br>• COVID-19 case management<br>• Dead body management

#	Author	Title	Document type	Key findings
	Ministry of Health			
8	Prime Minister's Office	Public Notice from the Prime Minister on Coronavirus Disease 2019.	Public notice	• Leadership involvement in COVID-19 preparedness and response (06th March 2020)
9	Mike Habinshuti YB, Nyamwasa D, et al.	COVID-19 Rwanda response updates.	Bulletin article	This report summarizes specific strategies that were used for the response to COVID-19:<br>• COVID-19 surveillance<br>• Testing with two additional testing sites, case management, IPC, risk communication and community engagement
Purposively identified WHO, Global fund, GFF, and other documents				
10	The Global Fund	The Impact of COVID-19 on HIV, TB and Malaria services and systems for health: A Snapshot from 502 Health Facilities Across Africa and Asia	Report	• Study includes Rwanda among the 32 countries (24 from Africa, 7 from Asia, Ukraine)<br>• All health facilities described change in attendance<br>• Examples of reduction in EBI coverage for children (as compared to 2019)<br>• COVID-19 has significantly affected health facility capacity to provide services:<br>o Only 17% of facilities in Africa had a sufficient operating budget<br>o Only 72% had the minimum stock level for the three diseases<br>• Various adaptive measures to mitigate this impact<br>• Adaptive measure for delivery of health services (most common: extended drug prescriptions – 71% of facilities)
11	Global Financing Facility	Preserve Essential Health Services during the COVID-19 Pandemic: Rwanda	Report	Methodology: derived modeling estimates using the Lives Saved Tool (LiST). Model assumes similar reduction in coverage of essential health services as the 2014 Ebola epidemic.<br>Estimations:<br>• 264,900 children without oral antibiotics for pneumonia<br>• 459,900 children without DPT vaccinations<br>• 93,300 women without access to facility-based deliveries<br>• 390,600 fewer women receiving family planning services.<br>• Child mortality in Rwanda could increase by 29% and maternal mortality by 23% over the next year

#	Author	Title	Document type	Key findings
12	The World Health Organization	Pulse survey on continuity of essential health services during the COVID-19 pandemic Interim report 27 August 2020	Interim Report	Methodology: Key informant surveys in among MOH officials in 105 countries (survey sent to 159 countries with a 66% response rate) to assess the impact of COVID-19 on 44 essential health services<br>• 80% of responding countries had created a package of EHS before the pandemic and 66% identified EHS that need to be maintained<br>• All countries (90%) reported some level of disruption, with LICs reporting more disruption<br>• Majority of the disruptions were partial (5-50% disruption in provision or use)<br>• All types of services were affected, with emergency services being the least affected (although 16 countries reported a disruption in ER)
13	World Health Organization	Second round of the national pulse survey on continuity of essential health services during the COVID-19 pandemic: January-March 2021	Interim Report	Second round of surveys in WHO countries – 135 respondents in this round, 94% of which reported disruptions in the delivery of EHS. Only 6% reported no disruption. On average, countries reported disruption in 38% of EHS. Slightly adapted methodology.<br>Disruption of services – Summary – RMNCAH and nutrition services:<br>• 35% of countries reported disruptions<br>• Most disrupted were family planning and contraceptive series & management of moderate and severe malnutrition (>40%).<br>• ANC and PNC (>33%), FBD (25%)<br>• Sick child visits reduced in 34% of countries (Though not clear if due to actual disruption of reduced chance of illness due to better hygiene, reduction in gatherings etc.)<br>• Disruption in immunization services was reported by 37% of countries.
14	World Health Organization [16]	COVID-19: Operational guidance for maintaining essential health services during an outbreak	Interim Guidance	Operational guidance on a set of immediate targeted actions that governments should consider to ensure the continuity of quality essential health services:<br>• Governance and coordination mechanisms to complement response protocols<br>• Identify context-specific essential services<br>• Optimize service delivery setting and platforms<br>• Establish effective patient flow<br>• Rapidly re-distribute health workforce capacity, including by re-assignment and task sharing (combination of increased work load and reduced HCWs will stress the health system)<br>• Identify mechanisms to maintain availability of essential medications, equipment, and supplies
Desk review of Medline/PubMed articles				

#	Author	Title	Document type	Key findings
15	Lazarus JV, Ratzan S, et al.	COVID-SCORE: A global survey to assess public perceptions of government responses to COVID-19 (COVID-SCORE-10).	Journal article	A cross-sectional study to validate the COVID-SCORE instrument and report results on public perception in 19 countries seriously affected by COVID-19.<br>• The COVID-SCORE was found both unidimensional and reliable.<br>• The mean country scores varied from 35.8 (Ecuador) to 80.5 (China) out of a maximum of 100 points.<br>• Country scores correlated with the people's levels of trust in government decisions.
16	D'Ambruoso L, Abbott P, and Binagwaho A.	Building back fairer in public health policy requires collective action with and for the most vulnerable in society.	Journal article	• The article highlighted that is not possible to build resilient and equitable health systems during crises such as pandemics.<br>• However, as preparatory strategies, it is required to build and maintain resilient and equitable health systems.
17	Chiwona-Karltun L, Amuakwa-Mensah F.	COVID-19: From health crises to food security anxiety and policy implications.	Journal article	• A mixed method study using qualitative data and secondary data analysis that assessed the effect of COVID-19 spread at local areas and its economic impact on food production.<br>• Concern about the local spread of COVID-19 and its economic impact increased the probability of food availability worries.<br>• The population most in need were supported by governments.
18	Adamu AA, Jalo RI, et al.	COVID-19 and routine childhood immunization in Africa: Leveraging systems thinking and implementation science to improve immunization system performance.	Journal article	The article highlighted that childhood vaccination is one of the routine health services that were disrupted by COVID-19 in Africa, increasing the risk of epidemics related to vaccine-preventable diseases. Therefore, policymakers need to urgently:<br>• Identify robust strategies specific to local context for scaling up the immunization services and mitigate COVID-19 impact on these services.<br>• This can be achieved through decision-making via systems thinking and implementation science which help to understand how childhood immunization, COVID-19, and its control strategies are interrelated.<br>• Thereafter, implementation science helps to guide how to identify areas for improvement and implement evidence-based interventions.
19	Ngamije J and Yadufashije C.	COVID-19 pandemic against mental health services for genocide survivors during commemoration week in Rwanda.	Journal article	• The article highlighted that the lockdown was ordered in Rwanda to minimize the COVID-19 transmission among the population. As the lockdown coincided with the commemoration week, mental health disorders, in addition to those that were exacerbated during the week, could have been worsened for genocide survivors because of decreased psychological support due to lockdown.

#	Author	Title	Document type	Key findings
20	Klingenberg C, Tembulkar SK, et al.	COVID-19 preparedness—a survey among neonatal care providers in low- and middle-income countries.	Journal article	This cross-sectional study assessed preparedness to COVID-19 response and found that there were significant COVID-19 challenges to neonatal care:<br>• LICs had worse shortages in equipment, staffing, equipment, and isolation during the pandemic than before.<br>• 26% of participants from SSA countries reported an increase in neonatal mortality due to infectious diseases other than COVID-19.<br>Implementation of clinical guidelines, specifically on isolation, cord clamping, and breastfeeding widely varied and was not in compliance with WHO guidelines.
21	Nachega Nachega JB, Grimwood A, et al.	From Easing Lockdowns to Scaling Up Community-based Coronavirus Disease 2019 Screening, Testing, and Contact Tracing in Africa-Shared Approaches, Innovations, and Challenges to Minimize Morbidity and Mortality.	Journal article	This viewpoint highlighted that a wide range of lockdown measures has helped to reduce the spread of COVID-19 but it results into economic problems in Africa.<br>• There are different effective measures for the pandemic control. These include efficient community screening, contact tracing, and testing; effective allocation of resources; behavior change interventions; and well-supported, trained, and protected community-based personnel.<br>• Rwanda implemented strategies based on existing pandemic challenges:<br>o Pool testing method for COVID-19 mass testing in response to limited laboratory capacity to run 1500 or more tests per day.<br>o Testing GeneXpert platform for phases 2 and 3 of the lockdown and establishing COVID-19 testing capacity using existing platforms at periphery level in response to long turnaround time of PCR results.<br>• Using GPS tracking devices for cross-border truck drivers in response to challenges with tracking the movement of the cross-border truck drivers.
22	McIntosh A, Bachmann M, et al.	Effect of COVID-19 lockdown on hospital admissions and mortality in rural KwaZulu-Natal, South Africa: interrupted time series analysis.	Journal article	The observational cohort study assessed impact of lockdown on hospital admissions.<br>• During level 5 lockdown (essential travels only, businesses closed, schools closed, and transport restricted), among children under-5, hospital admissions decreased compared to the period before COVID-19.<br>o The level 5 lockdown is prevented the most vulnerable population including children under-5 and those who were more severely ill from accessing hospital care services in rural areas.

#	Author	Title	Document type	Key findings
23	Murewanhema G and Makurumidze R.	Essential health services delivery in Zimbabwe during the COVID-19 pandemic: perspectives and Recommendations.	Journal article	The perspective reported that COVID-19 response measures including lockdown were imposed on 30 March 2020, 10 days after emergence of the 1st case of COVID-19 in Zimbabwe.<br>• This led to disruptions in healthcare services, people's movement of people, and supply chains. This led patients to delay in accessing and seeking healthcare.<br>• As leaders failed to provide appropriate PPEs, healthcare workers struggled to treat patients whose COVID-19 status is unknown. This resulted in restricting patients to receive treatment without COVID-19 test results.
24	Egger D, Miguel E, et al.	Falling living standards during the COVID-19 crisis: Quantitative evidence from nine developing countries.	Journal article	• Data analysis of 16 surveys in 9 countries including Rwanda found that there was reduced access to healthcare services including prenatal care and immunization services.
25	Aborode AT, David KB, et al.	Fighting COVID-19 at the Expense of Malaria in Africa: The Consequences and Policy Options.	Journal article	• This perspective highlighed that COVID-19 response measures hindered malaria control measures including IRS and distribution of LLINs in LMICs especially SSA as well as shifting to the production of COVID-19 RDTs from malaria RDTs production.
26	Hanney SR, Kanya L, et al.	How to strengthen a health research system: WHO's review, whose literature and who is providing leadership?	Journal article	The opinion piece clarifies that evidence synthesis for effective decision-making to strengthen in-country health research systems needs to conduct a thorough situation analysis, engage stakeholders and partners, maintain comprehensive strategies, critically assess health policies' impact, analyze the research system components, and identify strategies for addressing gaps.
27	McClure EM, Kinney MV, et al.	Impact of COVID-19 on maternal and child health.	Journal article	This correspondence piece responded to a paper that published estimates on COVID-19 impact on MCH services in LMICs using LiST analysis. As this paper had omitted stillbirths, McClure et al. (2020) recommended to also include stillbirths to have an estimate of additional deaths caused by COVID-19.
28	Hirschhorn LR, Smith JD, et al.	Integrating implementation science into covid-19 response and recovery.	Journal article	This editorial specified that efforts for COVID-19 response need to be based on implementation science approaches and maintain the delivery of evidence-based interventions for other health services with use of appropriate strategies adapted to the local context.

#	Author	Title	Document type	Key findings
29	Yoshikawa H, Wuermli AJ, et al.	Effects of the Global Coronavirus Disease-2019 Pandemic on Early Childhood Development: Short- and Long-Term Risks and Mitigating Program and Policy Actions.	Journal article	This commentary highlighted that one third of HIV+ pregnant women delayed in receiving ART, putting their children at risk of HIV infection. COVID-19 pandemic can affect children's development across the world through increased food insecurity and poverty, stress, loss of caregivers, and interrupted healthcare services.
30	Lazarus JV, Binagwaho A, et al.	Keeping governments accountable: the COVID-19 Assessment Scorecard (COVID-SCORE).	Journal article	The commentary reported that, during the pandemic, countries needed to strengthen health systems through adoption of strategies (ex. Task shifting and task sharing, telehealth technologies) to health service delivery, and engagement of different sectors.
31	Phillips DE, Bhutta ZA, et al.	Learning from Exemplars in Global Health: a road map for mitigating indirect effects of COVID-19 on maternal and child health.	Journal article	This commentary highlighted that: Countries need to consider domains of health systems domains (demand, delivery, resources, and social determinants) to minimize the pandemic effects through adopting strategies that have been successful in other countries and/or during other pandemics (strong national leadership, data-driven decision-making, strong community health program, and equity focus).
32	Villar J, Ariff S, et al.,	Maternal and Neonatal Morbidity and Mortality Among Pregnant Women With and Without COVID-19 Infection.	Journal article	The multinational cohort study found that:<br>• 49% of pregnant women with COVID-19 were overweight early in pregnancy compared to those without COVID-19 (40%)<br>• Pregnant women with COVID-19 were at increased risk of eclampsia/pre-eclampsia as opposed to those without COVID-19 (relative risk-RR: 1.76; 95% CI, 1.27-2.43).<br>Similarly, the pregnant women diagnosed with COVID-19 were at increased risk of infection (RR: 3.38; 95% CI, 1.63-7.01), maternal mortality (RR: 22.3; 95% CI, 2.88-172), preterm birth (RR: 1.59; 95% CI, 1.30-1.94), and admission in intensive care unit (RR: 5.04; 95% CI, 3.13-8.10).
33	Kimani RW, Maina R, et al.	Maternal and newborn care during the COVID-19 pandemic in Kenya: recontextualising	Journal article	• The review recognizes that COVID-19 caused disruptions in health services in Kenya and suggests strengthening community-based midwifery program to deliver maternity and newborn health services at community level, leading to avoiding unnecessary movements, decreased workload at the

#	Author	Title	Document type	Key findings
		the community midwifery model.		hospital level during the pandemic, minimized risk of spreading the pandemic to women and their babies.
34	Nachega JB, Kapata N, et al.	Minimizing the impact of the triple burden of COVID-19, tuberculosis and HIV on health services in sub-Saharan Africa.	Journal article	Based on modelling studies, COVID-19 predicted to cause disruptions in HIV and TB health services in SSA, resulting increase in associated mortality and morbidity in the next 5 years.
35	Nachega JB, Leisegang R, et al.	Mobile Health Technology for Enhancing the COVID-19 Response in Africa: A Potential Game Changer?	Journal article	The editorial reported that mHealth best practices that has been successful in many parts of Africa can be adapted and swiftly used to respond to COVID-19.
36	Tefera YG and Ayele AA.	Newborns and Under-5 Mortality in Ethiopia: The Necessity to Revitalize Partnership in Post-COVID-19 Era to Meet the SDG Targets.	Journal article	The commentary recommends considering development of strong partnerships during the post-COVID-19 period to achieve the SDG target of reducing neonatal and under-5 mortality.
37	Jewell BL, Mudimu E, et al.	Potential effects of disruption to HIV programmes in sub-Saharan Africa caused by COVID-19: results from multiple mathematical models.	Journal article	This mathematical modelling for SSA estimated that an interruption in ART supply to 50% of HIV+ people for 6 months would increase the risk of HIV-related deaths by 1.63 times and MTCT of HIV by 1.6 times for one year.
38	Umviligihozo G, Mupfumi L, et al.	Sub-Saharan Africa preparedness and response to the COVID-19 pandemic: A perspective of early career African scientists.	Journal article	This perspective provided recommendations for closing the gaps in COVID-19 preparedness and response:<br>• Sociocultural: implementation of online/technology platforms for health education, community education and engagement, and robust communication system to combat misinformation<br>• Biomedical: Skills development for health staff, creation of north-south/south-south partnerships for skills exchange, establishing local biotechnology capacity, evidence-based policies, building unbreakable supply chain systems, and strengthening health systems to make them uninterruptible.

#	Author	Title	Document type	Key findings
				Economic: resource mobilization for securing sustainable budget, strengthening micro-economies to increase GDP per capita, government support in establishing basic infrastructure, and sustainment of economic power for health programs.
39	Ahmed T, Rahman AE, et al.	The effect of COVID-19 on maternal newborn and child health (MNCH) services in Bangladesh, Nigeria and South Africa: call for a contextualized pandemic response in LMICs.	Journal article	The commentary highlights that, between March-May 2020 in South Africa, Bangladesh, and Nigeria, there was reduction in the utilization of EHS (MNCH- ANC, FP, and immunization) because of:<br>• Lockdown which caused fear to contract COVID-19, leading people to not seeking the EHS.<br>• Shifting resources to the pandemic response; shortage of resources.
40	Roberts L.	Measles is on the rise – and COVID-19 could make it worse.	Media special report	
41	Nshimyiryo A A, Barnhart DA, et al.	Barriers and coping mechanisms to accessing healthcare during the COVID-19 lockdown: a cross-sectional survey among patients with chronic diseases in rural Rwanda.	Journal article	A cross-sectional study among chronic care patients at 3 rural Rwandan districts (March to June 2020).<br>• Of 220 patient respondents, 44% reported at least one barrier to accessing healthcare. Barriers included lack of access to emergency care, lack of access to medication and skipping clinical appointments.<br>• Positive coping mechanisms to ensure continuation of care include: walking long distances during suspension of public transport, contacting clinicians via telephone for guidance or rescheduling appointments, and delegating someone else for medication pick-up.
42	Pierre G, Uwineza A, et al.	Attendance to HIV Antiretroviral Collection Clinic Appointments During COVID-19 Lockdown. A Single Center Study in Kigali, Rwanda.	Journal article	A cross-sectional study design conducted between March 21 to April 30, 2020at the University Teaching Hospital of Kigali (CHUK), ART clinic.<br>• ART patients from Kigali were more likely to attend scheduled ART clinic appointments (50%) within during the lockdown period compared to 35% among those living outside Kigali.<br>• There was an association. Those with WHO disease I (48%); attended scheduled ART collection clinic visit compared to 56% and 22% among those in WHO clinical stage 3 and 4, respectively.
43	Krisiunas E, Sibomana L.	Benefits of Technology in the Age of COVID-19 and Diabetes:	Case study	This is a case study. A WhatsApp group during this period created that included nurses in charge of diabetes from all district hospitals (42 hospitals) and Type I diabetes group leaders to gather information, challenges, and solutions.

#	Author	Title	Document type	Key findings
		Mobile Phones From a Rwanda Perspective.		• It is also being used as a fundraising tool, pay health insurance also known as mutuelle de santé and receive the information on the current status of medical supplies at each hospital and relay the message to senior health officials who then authorize a new delivery.
44	Umutesi G, Shyirambere C, et al.	Cancer care delivery innovations, experiences and challenges during the COVID-19 pandemic: The Rwanda experience.	Journal article	Four main interventions (March and June 2020) to:<br>• ensure access for continued cancer care;<br>• set up a drone-based drug re-fill community delivery;<br>• support continued cancer care among enrolled patients living in DRC and Burundi;<br>• provide social economic support to vulnerable patients.
45	Musanabaganwa C, Cubaka V, et al.	133 observed COVID-19 deaths in 10 months: unpacking lower than predicted mortality in Rwanda.	Journal article	• Between March to December 2020, 10 months after its first case, Rwanda recorded 10,316 cases and 133 COVID-19-related deaths; CFR=1.3%<br>• Effective COVID-19 response including infection control strategies, reliable reporting of cases and deaths, younger age contributed to the lower CFR
46	Uwizeyimana T, Hashim HT, et al.	Drug supply situation in Rwanda during COVID-19: issues, efforts and challenges.	Journal article	• COVID-19 disrupted drug supply system;<br>• Rwanda mitigated the effect;<br>• Investment to establish local manufacturing capacity is critical
47	Manirambona E, Uwizeyimana T, et al.	Impact of the COVID-19 pandemic on the food rations of refugees in Rwanda.	Journal article	• Efforts to ensure refugees can afford the basic necessities needed for life in Rwanda is required; the new policy to reduce refugees' food rations would affect the livelihood during the period of COVID-19.
48	Iradukunda PG, Pierre G, et al.	Knowledge, Attitude, and Practice Towards COVID-19 Among People Living with HIV/AIDS in Kigali, Rwanda.	Journal article	A cross-sectional KPA survey among PLHIV towards COVID-19 prevention and control measures. 97% obtained a high knowledge score; 26% had a poor attitude score; 90% had high practice.
49	Karim N, Jing L et al.	Lessons Learned from Rwanda: Innovative Strategies for Prevention and	Viewpoint	• Rwandan pandemic preparedness started in January 2020; RBC coordinated the effort.<br>• Strategies included: a six month National COVID-19 Preparedness and Response Plan that detailed protocols for early testing, contact tracing, CHWs outreach program, disseminating of information using drones, robots for screening and inpatient care,

#	Author	Title	Document type	Key findings
		Containment of COVID-19.		and official communications to combat misinformation and timely communication
50	Wanyana D, Wong R, et al.	Rapid assessment on the utilization of maternal and child health services during COVID-19 in Rwanda.	Journal article	This cross-sectional quantitative study reported the change in the utilization of the MCH services during COVID-19:<br>• ANC first standard visits from 0.49 to 0.45 (P = 0.042)<br>• FBD from 0.99 to 0.89 (P = 0.004)<br>• PNC 4 visit for baby from 0.43 to 0.50 (P = 0.005); PNC 4 visit for mother from 0.44 to 0.51 (P = 0.011)<br>• BCG from 1.06 to 0.95 (P = 0.002)<br>• polio zero from 0.97 to 0.86 (P = 0.001); polio 1 from 0.97 to 0.89 (P = 0.008); polio 2 from 0.95 to 0.88 (P = 0.008)<br>• DTP_HepB_Hib 1 from 0.97 to 0.89 (P = 0.007); DTP_HepB_Hib 2 from 0.95 to 0.88 (P = 0.007)<br>• pneumococcus 1 from 0.97 to 0.89 (P = 0.007); pneumococcus 2 from 0.95 to 0.88 (P = 0.007)<br>• rotavirus 1 from 0.98 to 0.89 (P = 0.006); rotavirus 2 from 0.95 to 0.88 (P = 0.009)
51	Ndishimye Pacifique NM, Hitimana N, et al.	Knowledge, attitudes and preventive practices towards COVID-19 among frontline healthcare workers in Rwanda.	Bulletin article	Descriptive cross-sectional study which assessed knowledge, attitudes and preventive practices towards COVID-19 among frontline healthcare workers in Rwanda. Key findings were:<br>• 100% of the respondents were aware of COVID-19 measures such as self-isolation and quarantine in case of COVID-19 symptoms or infection.<br>• 95% of the respondents adopted COVID-19 prevention measures such hand hygiene and social distancing.

1. Rwanda Biomedical Center. Rapid Assessment for Continuity of Minimum Essential Reproductive Maternal, Newborn, Child and Adolescent Health and Nutrition services in the Context of COVID-19 Response in Rwanda. Accessed March 31, 2021.
2. Ntazimira C BP, Mbituyumuremyi A. The Impact of COVID 19 on Malaria Program in Rwanda: Rapid Assessment of Knowledge, Attitudes, Perspectives on COVID 19, and Situation of Malaria Services Provision in Three Districts in Rwanda. 2021. Accessed March 31, 2021. https://www.rbc.gov.rw/fileadmin/user_upload/annoucement/The Impact of COVID 19 on Malaria Program in Rwanda.pdf
3. UNICEF. Rwanda COVID-19 situation report no 06. Reporting period: 1 October to 30 November 2020. https://www.unicef.org/rwanda/media/2666/file/2020-UNICEF-COVID-Situation-Report-October-November.pdf
4. Hartnett Bridget NJ, Nsanzabaganwa Christian, Ndishimye Pacifique, Hitimana Nadia, Byiringiro Fidele, Nyamusore Jose, et al. COVID-19 Rwanda response updates March 14 – April 25, 2020. *Rw Public Health Bul* March 2020; **2**(1).
5. Rwanda Biomedical Center & Rwanda Ministry of Health. Standard Operating Procedures for Preparedness and Response to COVID-19 outbreak. 2021. Accessed March 31, 2021.

https://rbc.gov.rw/fileadmin/user_upload/annoucement/SOP%20for%20preparedness%20and%20response%20to%20Covid-19.pdf
6. Rwanda Biomedical Center & Rwanda Ministry of Health. Coronavirus disease 2019, National preparedness and response plan. Accessed March 31, 2021. https://www.rbc.gov.rw/fileadmin/user_upload/annoucement/Coronavirus%20Disease%202019,%20National%20Preparedness%20and%20Response%20Plan.pdf.
7. Rwanda Biomedical Center & Rwanda Ministry of Health. COVID-19 clinical Management guidelines. Accessed March 31, 2021. https://www.moh.gov.rw/fileadmin/user_upload/Moh/Publications/Guidelines_Protocols/Covid_-19_Clinical_Management_Guideline.pdf
8. Prime Minister's Office. Public Notice from the Prime Minister on Coronavirus Disease 2019. 2020. Accessed March 31, 2021. https://www.primature.gov.rw/index.php?id=131&tx_news_pi1%5Bnews%5D=900&tx_news_pi1%5Bcontroller%5D=News&tx_news_pi1%5Baction%5D=detail&cHash=49468fe2d77a94e5a7f1653daddc6df3
9. Mike Habinshuti YB, Daniel Nyamwasa, Clarisse Musanabaganwa, Pacifique Ndishimye, Nadia Hitimana, Theophile Dushime, et al. COVID-19 Rwanda response updates. *Rw Public Health Bul* 2020; **2**(2).
10. The Global Fund. The Impact of COVID-19 on HIV, TB and Malaria Services and Systems for Health - Updates - The Global Fund to Fight AIDS, Tuberculosis and Malaria; 2021. Accessed June 10, 2021. https://www.theglobalfund.org/en/updates/other-updates/2021-04-13-the-impact-of-covid-19-on-hiv-tb-and-malaria-services-and-systems-for-health/.
11. Global Financing Facility. Preserve EHS during the COVID-19 pandemic, Rwanda. Undated. Accessed June 9, 2021. https://www.globalfinancingfacility.org/sites/gff_new/files/documents/Rwanda-Covid-Brief_GFF.pdf.
12. World Health Organization. Pulse Survey on Continuity of Essential Health Services during the COVID-19 Pandemic: Interim Report.; 2020. Accessed June 9, 2021. https://www.who.int/publications/i/item/WHO-2019-nCoV-EHS_continuity-survey-2020.1.
13. World Health Organization. Second Round of the National Pulse Survey on Continuity of Essential Health Services during the COVID-19 Pandemic: January-March 2021.; 2021. Accessed June 10, 2021. https://www.who.int/publications/i/item/WHO-2019-nCoV-EHS-continuity-survey-2021.1.
14. World Health Organization. COVID-19: Operational Guidance for Maintaining Essential Health Services during an Outbreak: Interim Guidance.; 2020. Accessed June 10, 2021. https://iris.who.int/handle/10665/331561
15. Lazarus JV, Ratzan S, Palayew A, et al. COVID-SCORE: A global survey to assess public perceptions of government responses to COVID-19 (COVID-SCORE-10). *PLoS One* 2020; **15**(10): e0240011.
16. D'Ambruoso L, Abbott P, Binagwaho A. Building back fairer in public health policy requires collective action with and for the most vulnerable in society. *BMJ Glob Health* 2021; **6**(3).
17. Chiwona-Karltun L, Amuakwa-Mensah F, Wamala-Larsson C, et al. COVID-19: From health crises to food security anxiety and policy implications. *Ambio* 2021; **50**(4): 794-811.
18. Adamu AA, Jalo RI, Habonimana D, Wiysonge CS. COVID-19 and routine childhood immunization in Africa: Leveraging systems thinking and implementation science to improve immunization system performance. *Int J Infect Dis* 2020; **98**: 161-5.
19. Ngamije J, Yadufashije C. COVID-19 pandemic against mental health services for genocide survivors during commemoration week in Rwanda. *Heliyon* 2020; **6**(10): e05229.
20. Klingenberg C, Tembulkar SK, Lavizzari A, et al. COVID-19 preparedness-a survey among neonatal care providers in low- and middle-income countries. *J Perinatol* 2021: 1-10.
21. Nachega JB, Grimwood A, Mahomed H, et al. From Easing Lockdowns to Scaling Up Community-based Coronavirus Disease 2019 Screening, Testing, and Contact Tracing in Africa-Shared

Approaches, Innovations, and Challenges to Minimize Morbidity and Mortality. *Clin Infect Dis* 2021; **72**(2): 327-31.
22. McIntosh A, Bachmann M, Siedner MJ, Gareta D, Seeley J, Herbst K. Effect of COVID-19 lockdown on hospital admissions and mortality in rural KwaZulu-Natal, South Africa: interrupted time series analysis. *BMJ Open* 2021; **11**(3): e047961.
23. Murewanhema G, Makurumidze R. Essential health services delivery in Zimbabwe during the COVID-19 pandemic: perspectives and recommendations. *Pan Afr Med J* 2020; **35**(Suppl 2): 143.
24. Egger D, Miguel E, Warren SS, et al. Falling living standards during the COVID-19 crisis: Quantitative evidence from nine developing countries. *Sci Adv* 2021; **7**(6).
25. Aborode AT, David KB, Uwishema O, et al. Fighting COVID-19 at the Expense of Malaria in Africa: The Consequences and Policy Options. *Am J Trop Med Hyg* 2021; **104**(1): 26-9.
26. Hanney SR, Kanya L, Pokhrel S, Jones TH, Boaz A. How to strengthen a health research system: WHO's review, whose literature and who is providing leadership? *Health Res Policy Syst* 2020; **18**(1): 72.
27. McClure EM, Kinney MV, Leisher SH, et al. Impact of COVID-19 on maternal and child health. *Lancet Glob Health* 2020; **8**(10): e1258.
28. Hirschhorn LR, Smith JD, Frisch MF, Binagwaho A. Integrating implementation science into covid-19 response and recovery. *Bmj* 2020; **369**: m1888.
29. Yoshikawa H, Wuermli AJ, Britto PR, et al. Effects of the Global Coronavirus Disease-2019 Pandemic on Early Childhood Development: Short- and Long-Term Risks and Mitigating Program and Policy Actions. *J Pediatr* 2020; **223**: 188-93.
30. Lazarus JV, Binagwaho A, El-Mohandes AAE, et al. Keeping governments accountable: the COVID-19 Assessment Scorecard (COVID-SCORE). *Nat Med* 2020; **26**(7): 1005-8.
31. Phillips DE, Bhutta ZA, Binagwaho A, et al. Learning from Exemplars in Global Health: a road map for mitigating indirect effects of COVID-19 on maternal and child health. *BMJ Glob Health* 2020; **5**(7).
32. Villar J, Ariff S, Gunier RB, et al. Maternal and Neonatal Morbidity and Mortality Among Pregnant Women With and Without COVID-19 Infection: The INTERCOVID Multinational Cohort Study. *JAMA Pediatr* 2021.
33. Kimani RW, Maina R, Shumba C, Shaibu S. Maternal and newborn care during the COVID-19 pandemic in Kenya: re-contextualising the community midwifery model. *Hum Resour Health* 2020; **18**(1): 75.
34. Nachega JB, Kapata N, Sam-Agudu NA, et al. Minimizing the impact of the triple burden of COVID-19, tuberculosis and HIV on health services in sub-Saharan Africa. *Int J Infect Dis* 2021.
35. Nachega JB, Leisegang R, Kallay O, Mills EJ, Zumla A, Lester RT. Mobile Health Technology for Enhancing the COVID-19 Response in Africa: A Potential Game Changer? *Am J Trop Med Hyg* 2020; **103**(1): 3-5.
36. Tefera YG, Ayele AA. Newborns and Under-5 Mortality in Ethiopia: The Necessity to Revitalize Partnership in Post-COVID-19 Era to Meet the SDG Targets. *J Prim Care Community Health* 2021; **12**: 2150132721996889.
37. Jewell BL, Mudimu E, Stover J, et al. Potential effects of disruption to HIV programmes in sub-Saharan Africa caused by COVID-19: results from multiple mathematical models. *Lancet HIV* 2020; **7**(9): e629-e40.
38. Umviligihozo G, Mupfumi L, Sonela N, et al. Sub-Saharan Africa preparedness and response to the COVID-19 pandemic: A perspective of early career African scientists. *Wellcome Open Res* 2020; **5**: 163.
39. Ahmed T, Rahman AE, Amole TG, et al. The effect of COVID-19 on maternal newborn and child health (MNCH) services in Bangladesh, Nigeria and South Africa: call for a contextualised pandemic response in LMICs. *Int J Equity Health* 2021; **20**(1): 77.
40. Roberts L. Why measles deaths are surging - and coronavirus could make it worse. *Nature* 2020; **580**(7804): 446-7.

41. Nshimyiryo A, Barnhart DA, Cubaka VK, et al. Barriers and coping mechanisms to accessing healthcare during the COVID-19 lockdown: a cross-sectional survey among patients with chronic diseases in rural Rwanda. *BMC Public Health* 2021; **21**(1): 704.
42. Pierre G, Uwineza A, Dzinamarira T. Attendance to HIV Antiretroviral Collection Clinic Appointments During COVID-19 Lockdown. A Single Center Study in Kigali, Rwanda. *AIDS Behav* 2020; **24**(12): 3299-301.
43. Krisiunas E, Sibomana L. Benefits of Technology in the Age of COVID-19 and Diabetes. . .Mobile Phones From a Rwanda Perspective. *J Diabetes Sci Technol* 2020; **14**(4): 748-9.
44. Umutesi G, Shyirambere C, Bigirimana JB, et al. Cancer care delivery innovations, experiences and challenges during the COVID-19 pandemic: The Rwanda experience. *J Glob Health* 2021; **11**: 03067.
45. Musanabaganwa C, Cubaka V, Mpabuka E, et al. One hundred thirty-three observed COVID-19 deaths in 10 months: unpacking lower than predicted mortality in Rwanda. *BMJ Glob Health* 2021; **6**(2).
46. Uwizeyimana T, Hashim HT, Kabakambira JD, et al. Drug supply situation in Rwanda during COVID-19: issues, efforts and challenges. *J Pharm Policy Pract* 2021; **14**(1): 12.
47. Manirambona E, Uwizeyimana T, Uwiringiyimana E, Reddy H. Impact of the COVID-19 pandemic on the food rations of refugees in Rwanda. *Int J Equity Health* 2021; **20**(1): 107.
48. Iradukunda PG, Pierre G, Muhozi V, Denhere K, Dzinamarira T. Knowledge, Attitude, and Practice Towards COVID-19 Among People Living with HIV/AIDS in Kigali, Rwanda. *J Community Health* 2021; **46**(2): 245-50.
49. Karim N, Jing L, Lee JA, et al. Lessons Learned from Rwanda: Innovative Strategies for Prevention and Containment of COVID-19. *Ann Glob Health* 2021; **87**(1): 23.
50. Wanyana D, Wong R, Hakizimana D. Rapid assessment on the utilization of maternal and child health services during COVID-19 in Rwanda. *Public Health Action* 2021; **11**(1): 12-21.
51. Ndishimye Pacifique NM, Hitimana N, Turatsinze D, Nahayo E, Byiringiro Fidele HM, Nyamusore J, et al. Knowledge, attitudes and preventive practices towards COVID-19 among frontline healthcare workers in Rwanda. *Rw Public Health Bul* March 2020; **2**(1).
